# Fabrication of Al-Al_3_Ti/Ti_3_Al Functionally Graded Materials under a Centrifugal Force

**DOI:** 10.3390/ma3094639

**Published:** 2010-09-08

**Authors:** Shimaa El-Hadad, Hisashi Sato, Eri Miura-Fujiwara, Yoshimi Watanabe

**Affiliations:** 1 Department of Engineering Physics, Electronics and Mechanics, Nagoya Institute of Technology, Gokiso-cho, Showa-ku, Nagoya 466-8555, Japan; E-Mails: sato.hisashi@nitech.ac.jp (H.S.); emiura@nitech.ac.jp (E.M.-F.); yoshimi@nitech.ac.jp (Y.W.); 2 Department of Manufacturing Technology, Central Metallurgical Research & Development Institute, P.O.BOX 87, Helwan, Cairo, Egypt

**Keywords:** functionally graded materials (FGMs), centrifugal method (CM), reaction mixed-powder method

## Abstract

Fabrication of Al-Al_3_Ti functionally graded materials (FGMs) under the centrifugal force has recently attracted some attention. The controlled compositional gradient of the fabricated FGMs, the low cost of the process, and the good mold filling, are the main advantages of the centrifugal method (CM). Using the conventional CM techniques such as the centrifugal solid-particle method and centrifugal *in-situ* method, FGMs rings with gradually distributed properties could be achieved. As a more practical choice, the centrifugal mixed-powder method (CMPM) was recently proposed to obtain FGMs containing nano-particles selectively dispersed in the outer surface of the fabricated parts. However, if a control of the particles morphology, compound formulas or sizes, is desired, another CM technique is favored. As a development of CMPM, our novel reaction centrifugal mixed-powder method (RCMPM) has been presented. Using RCMPM, Al‑Al_3_Ti/Ti_3_Al FGMs with good surface properties and temperature controlled compositional gradient could be achieved. In this short review, this novel method will be discussed in detail and the effect of RCMPM processing temperature on the reinforcement particles morphology, size and distribution through the fabricated samples, will be reviewed.

## 1. Introduction 

Functionally graded materials (FGMs) are currently receiving a great degree of interest due to its special merits. This group of advanced multifunctional composites has a special volume fraction distribution of the reinforcements phase(s) (or dispersoids) which vary smoothly from one side to another in the fabricated part [1,2,3,4,5,6,7]. This is achieved by using reinforcements with different properties, sizes, and shapes, as well as by interchanging the functions of the reinforcement and matrix phases in a continuous manner. The result is a microstructure bearing continuous changes in thermal and mechanical properties at the macroscopic or continuum scale [8,9]. Several FGM fabrication methods have been proposed, such as chemical vapor deposition, plasma spray technique and various powder metallurgy techniques. However, it has been difficult to produce relatively large FGM components by most fabrication methods. In addition, those methods require relatively new techniques and expensive fabrication equipment [3,10]. 

Fabrication of FGMs based on mass/heat transport phenomenon includes the self-propagating synthesis and gravity or centrifugal segregation methods were reported by Suresh and Mortensen [10]. Fukui and Watanabe [3,4,5,6,7] have suggested the centrifugal method (CM) to disperse the reinforcement particles in a ductile metal matrix. In this method, centrifugal force is applied to a mixture of molten metal and dispersed material, such as ceramic powder or intermetallics compounds, leads to the formation of a desired composition gradient. The gradient is controlled mainly by the difference in density between the matrix and the dispersed material. 

**Figure 1:**
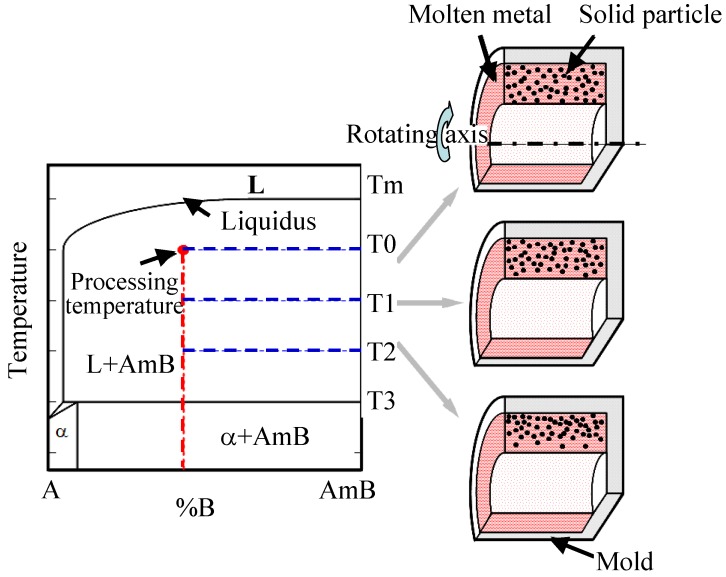
The schematic illustration of CSPM [15].

According to their series of works, Watanabe *et al.* [11,12,13] have classified the fabrication of FGMs using the centrifugal method into two categories based on the relation between the processing temperature and the liquidus temperature of the master alloy. If the liquidus temperature of the master alloy is significantly higher than the processing temperature as shown in Figure 1, the dispersed phase remains solid in a liquid matrix during the centrifugal casting. This situation is similar to ceramic‑dispersed FGMs, and this method is referred to as a centrifugal solid-particle method (CSPM) [14,15]. 

**Figure 2:**
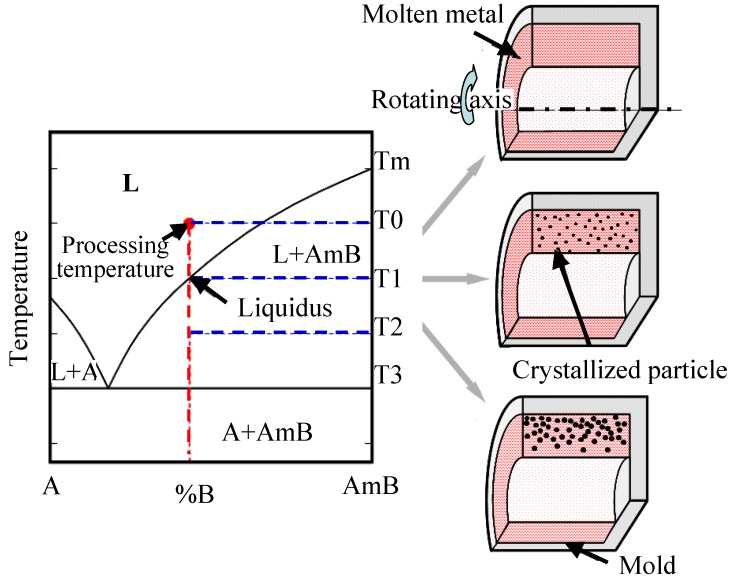
The schematic illustration of CISM [15].

On the other hand, if the liquidus temperature of the master alloy is lower than the processing temperature as shown in Figure 2, centrifugal force can be applied during the solidification of both the dispersed phase and the matrix. This solidification is similar to the production of *in situ* composites using the crystallization phenomena, and this method is, therefore, named a centrifugal *in situ* method (CISM) [16,17].

**Figure 3:**
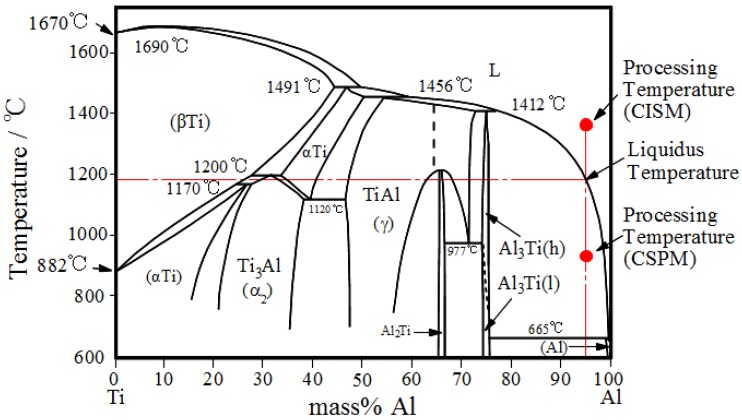
Al-Ti phase diagram [18].

Figure 3 shows Al-Ti phase diagram. The Al- 5 mass % Ti alloy as an example has a liquidus temperature of about 1200 °C. If the process is carried out below this temperature, then Al_3_Ti particles would stay solid in a liquid Al matrix during mold spinning (CSPM). On the contrary, performing CM at temperatures higher than 1200 °C would include melting of Al_3_Ti particles; thus both of the second phases and Al melt would solidify under the centrifugal force (CISM).

**Figure 4:**
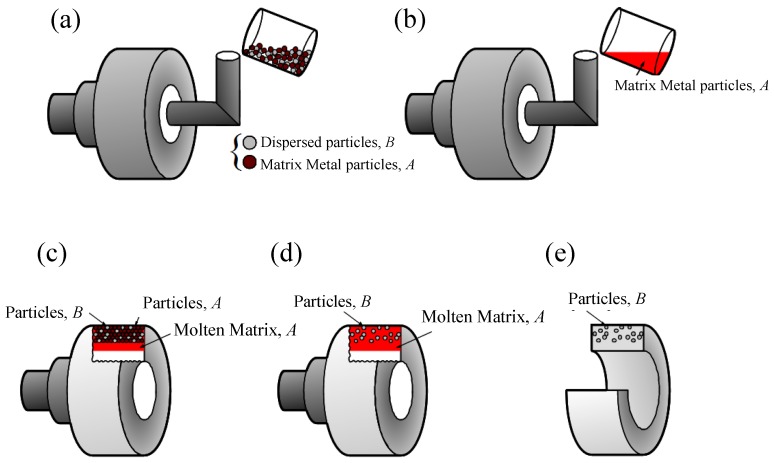
The schematic description of CSPM [19].

As a more developed CM process, the centrifugal mixed-powder method (CMPM) was recently proposed by Watanabe *et al*. [19,20]. As a first step of the CMPM, a powder mixture of dispersion-particles, *B*, and matrix metal particles, *A*, is inserted into a spinning mold (Figure 4 (a)). Then, matrix metal ingot, *A*, is melted and poured into the spinning mold with powder mixture *A + B*, as shown in Figure 4 (b). As a result, the molten matrix metal, *A*, penetrates into the space between the particles by the centrifugal force pressure as shown in Figure 4 (c). At the same time, powder of matrix metal, *A*, is melted by the heat from the molten matrix poured from a crucible (Figure 4 (d)). Finally, an FGM ring with dispersion-particles, *B*, distributed on its surface, can be obtained as shown in Figure 4 (e). When comparing the CMPM technique to the novel reaction (RCMPM), the main difference is that formation of the reinforcement particles in RCMPM occurs during the mold spinning by reaction. Namely, particles *B* and metal matrix *A* can be reacted as m*A* + n*B* = *A_m_B_n_*. Therefore, the processing temperature at which the powder/molten metal matrix react to form the reinforcements is the essential point in RCMPM. Recently, RCMPM has been applied for the Al-Ti system [21].

In the current review, we focus on the fabrication of Al-Al_3_Ti/Ti_3_Al FGMs using a new reaction centrifugal-mixed powder method (RCMPM). Since our developed method is based on both the CSPM and CISM conventional techniques, a brief description about these CM processes will be presented first to reveal the idea behind our new method. Some reported effects of the centrifugal casting processing temperature on the structural properties of the fabricated FGMs will be given to make a background for the novel RCMPM. Finally the experiments and results of our novel RCMPM will be explained.

## 2. Al-Al_3_Ti FGM Fabricated by CSPM

**Figure 5:**
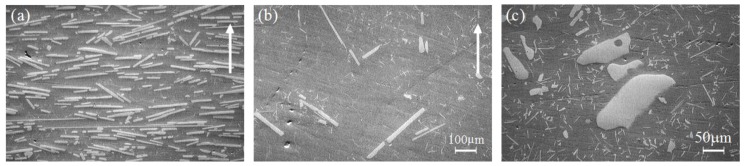
Microstructure of Al-Al_3_Ti CSPM-FGM [15]. (**a**) Images were taken on a plane perpendicular to the rotation axis in the outer; and (**b**) inner regions; (**c**) was taken on a plane perpendicular to the centrifugal force direction in the outer region of the ring.

Figure 5 shows the typical microstructure of Al-Al_3_Ti FGM fabricated by CSPM under *G* = 30 [15]. Figure 5 (a) and Figure 5 (b) are taken on a plane perpendicular to the rotation axis in outer and inner regions, respectively, and Figure 5 (c) is taken on a plane perpendicular to the centrifugal force direction in outer region of the ring. The direction of centrifugal force in (a) and (b) is indicated by an arrow. It can be seen from these figures that the volume fraction of Al_3_Ti particles decreases toward the inner side of the ring. This is because the densities of Al_3_Ti and molten aluminum at 700 °C are 3.4 Mg/m^3^ and 2.37 Mg/m^3^ [22], respectively. It must be noted here that in the outer region, most of platelets are oriented with their planes nearly normal to the radial direction (the centrifugal force direction) as shown in Figure 5 (a), while no orientation effect was observed in the inner region of the ring, as shown in Figure 5 (b).

**Figure 6:**
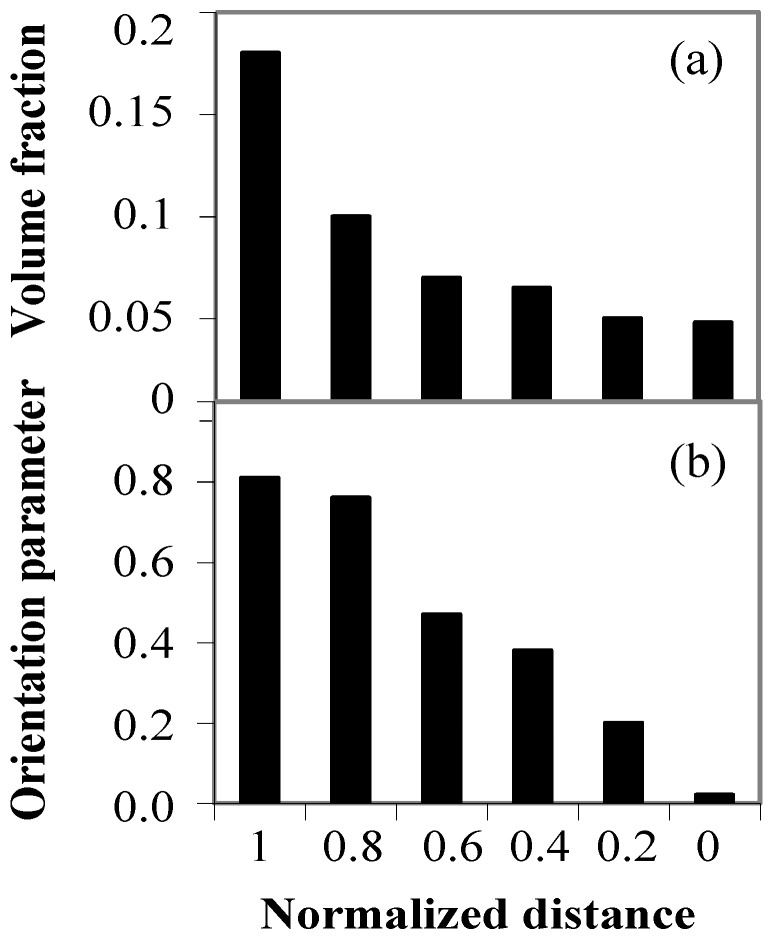
(**a**) Volume fraction; and (**b**) orientation parameter; distribution of Al_3_Ti inside Al-Al_3_Ti FGM [23].

Figure 6 (a) shows the distribution of Al_3_Ti platelets volume fraction in the FGM fabricated by CSPM under *G* = 30 observed on the plane perpendicular to the centrifugal force direction. The abscissa in this figure represents the position of normalized thickness; *i.e.*, 1.0 is the outer surface and 0.0 is the inner surface of the ring. As can be seen, the volume fraction of the Al_3_Ti platelets decreases toward the inner region of the ring. 

In order to describe the degree of platelet particles orientation, the following Hermans orientation parameter, *fp* [5,24] was calculated.

*fp* = 2<cos^2^*θ*> − 1 (1)

where the trigonometric average is,

π/2 <cos^2^*θ*> = ∫cos^2^*θ*n(*θ*)d*θ* −π/2(2)

The term n(*θ*) is the orientation distribution function which specifies the fraction of platelets within the angular element d*θ*. The parameter *fp* becomes 0 for a random distribution of platelets, and it becomes 1 for perfect alignments with their planes perpendicular to the radial direction. Intermediate values of this parameter correspond to partial states of orientation. The position dependency of the orientation parameter along the centrifugal force direction in the FGM is shown in Figure 6 (b). It is clearly seen that the orientation parameter decreases with the normalized thickness, and the FGMs have an orientation parameter gradient. Thus, we can fabricate Al-Al_3_Ti platelets FGM, in which the orientation of Al_3_Ti platelets, as well as the volume fraction of them, is distributed in a graded manner.

**Figure 7:**
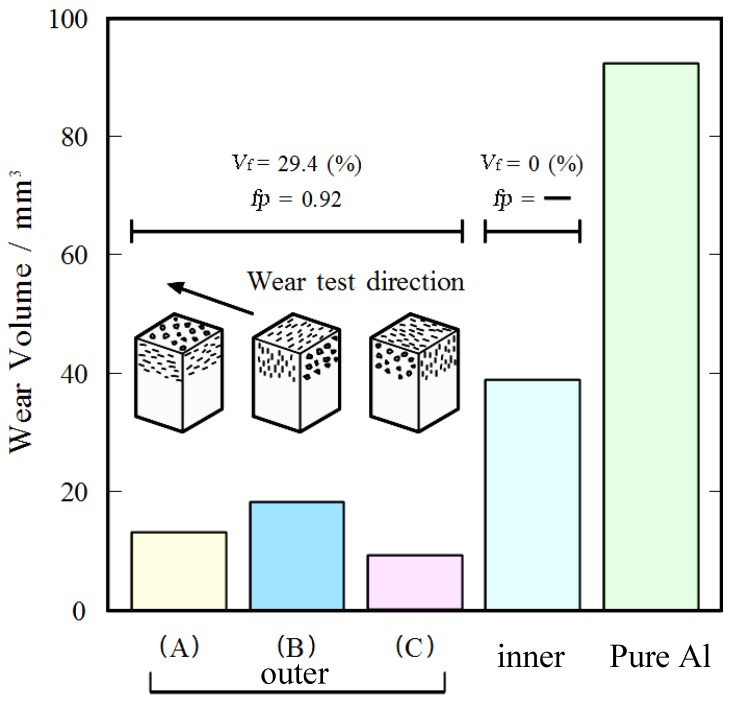
Wear volumes of the FGM fabricated at *G* = 50 [23].

Therefore, the positional and directional dependencies of wear properties might be expected in Al‑Al_3_Ti FGM. The wear tests are then carried out for different directions and positions, and results are shown in Figure 7, where the FGM was fabricated by CSPM under *G* = 50. The result for a pure Al specimen made by the same process is also shown in the figure for comparison. The wear volumes in the Al-Al_3_Ti FGMs are much smaller than those of pure Al. It is also seen from these figures that the wear volume at the ring’s outer region is smaller than that at the inner region. It should be mentioned here that in the case of the ring's outer region, considerable anisotropy exists in the wear volume among the three platelet orientations tested, where direction (B) shows the worst wear resistance among the three orientations. In contrast, directions (A) and (C) show relatively better wear resistance, and the wear volume of direction (C) is slightly smaller than that of direction (A). Although not presented here, anisotropic wear resistance was not observed at the ring’s inner region

## 3. Al-Al_3_Ti FGM Fabricated by CISM 

**Figure 8:**
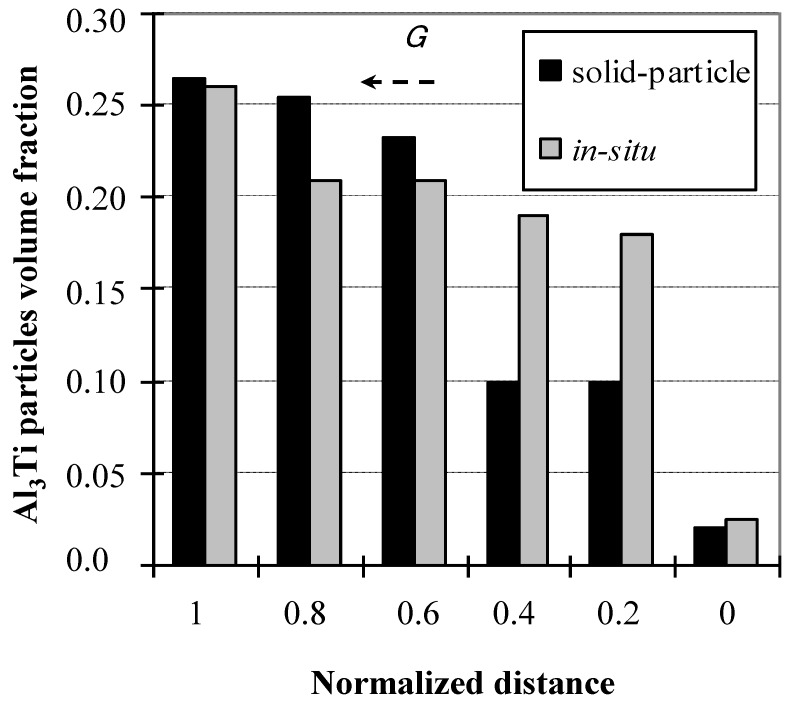
Volume fraction distribution of Al_3_Ti along the thickness of the Al-Al_3_Ti CM-FGM ring [25].

In order to understand the difference between the CISM-FGMs and CSPM-FGMs properties, Al-Al_3_Ti FGMs rings containing Al_3_Ti platelet particles distributed in Al matrix are given as an example. Figure 8 is the volume fraction distribution of Al_3_Ti particles in Al-Al_3_Ti FGM ring prepared in our previous work [25]. These ring samples were processed at 800 °C by the centrifugal solid-particle method and at 1600 °C by the centrifugal *in-situ* method. The normalized distance in this figure starts at (1.0) and ends at (0.0) which respectively represents the outer and inner surfaces of the fabricated ring. It is observed that the interior and inner regions of the fabricated rings in case of CSPM are almost deplete of Al_3_Ti particles [25]. On the other hand, in the case of FGMs fabricated by CISM, the compositional gradient of the particles is affected by the solidification process under the centrifugal force [16,17]. The presence of a considerable amount of particles in the interior regions in Figure 8 is an indication of this phenomenon. This is because the centrifugal force is applied to a completely liquid phase in the case of CISM, whereby the second phase intermetallics crystallize from the liquid phase during the solidification process [11]. As a result, the formation mechanism in CISM occurs due to the difference in density between the primary (Al_3_Ti) crystals and the liquid matrix [11]. Accordingly, the particles migration rate was slow and the compositional gradient gentle, due to the low diffusivity of Ti in Al [26] as shown in Figure 8.

**Figure 9:**
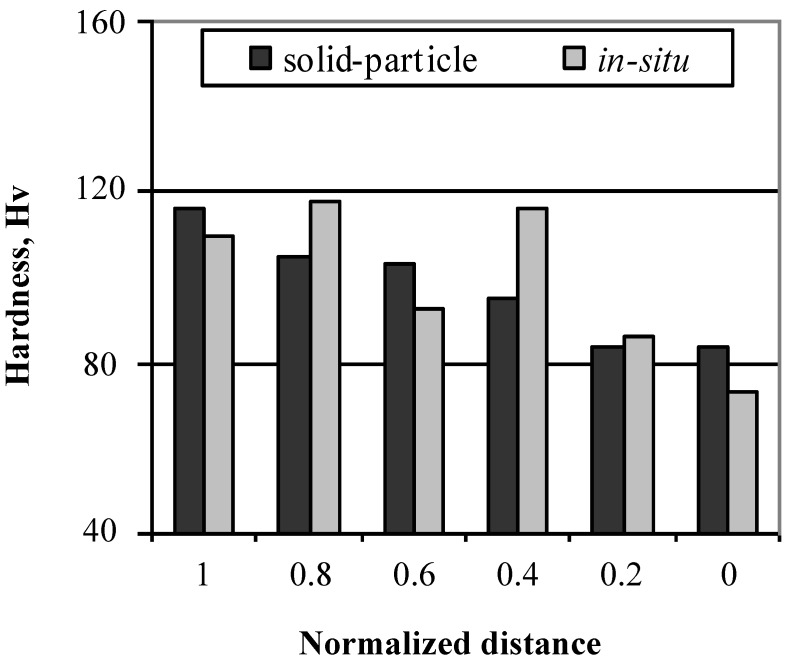
Hardness distribution gradient in Al-Al_3_Ti CM-FGM [25].

Since the obtained compositional gradient is different in the two CM techniques, it is expected that the mechanical properties distribution will follow the same trend as well. Figure 9 is the hardness distribution gradient obtained in the Al-Al_3_Ti FGMs presented in Figure 8. It is obvious that processing the FGMs at high temperatures (CISM) results in slower distribution of the property compared to the steep trend obtained in the case of low temperature (CSPM) processing. 

Here, we should note that the ingots used in the reported studies of the two CM categories include the reinforcement particles distributed randomly in the matrix. On this basis, the Al_3_Ti particles in the case of CSPM, and the Al_3_Ti primary crystals in CISM, will be homogeneously distributed in the melt before being selectively distributed in the mold under the centrifugal force. Whereas in the case of RCMPM, pure Ti will be added as powder which then reacts with Al melt; therefore the Al/Ti reaction and the resultant compounds will be essentially dependent on the processing temperature. 

## 4. Al-Al_3_Ti/Ti_3_Al FGM Fabricated by RCMPM

**Figure 10:**
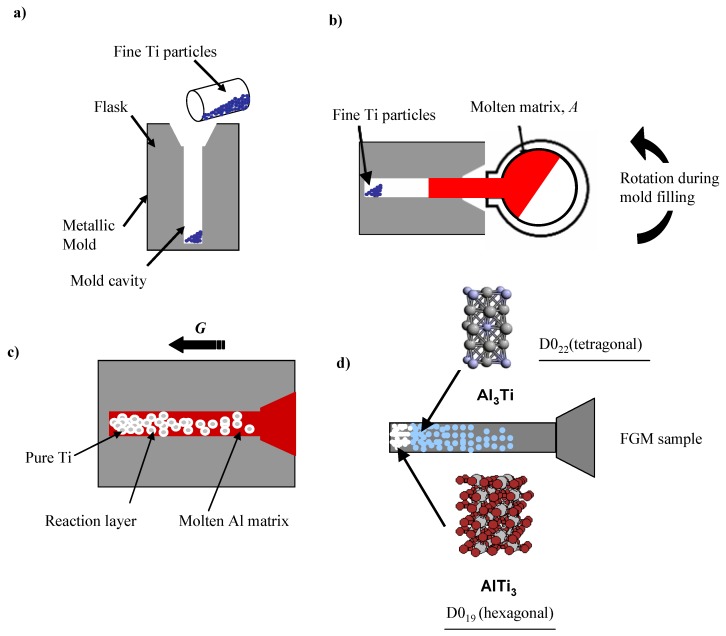
A schematic illustration showing the process of the centrifugal mixed-powder method [21].

The RCMPM-FGMs were fabricated using an investment centrifugal casting machine; a mold cylindrical cavity of 8 × 50 mm size is prepared by the investment technique [27]. The schematics of FGMs fabrication procedures using RCMPM are shown in Figure 10. The target master alloy is Al‑10 mass %Ti. The process can be described in three steps: first; the fine Ti powder (45 μm size) is placed into the mold cavity as shown in (Figure 10(a)).The pure Al metal is then inserted in the melting crucible surrounded by the induction coil. The casting temperatures varied between 1150 °C and 1450 °C and the liquidus temperature of the master alloy is around 1153 °C, as estimated from Al‑Ti phase diagram of Figure 3.When the Al melt reaches the required temperature, the centrifugal force is applied while the Al melt fills the mold cavity penetrating into the spaces between the Ti particles as illustrated in Figure 10(b). As a result, a partial/complete melting of the Ti particles occurs thus causing Al/Ti reaction layer on the Ti particles surface, as shown in Figure 10(c). Finally, Al_x_Ti intermetallic compounds are formed and the compositional gradient would vary through the sample length depending on the processing temperature (Figure 10(d)).The applied centrifugal force magnitude used in the experiments was *G* = 80.

**Figure 11:**
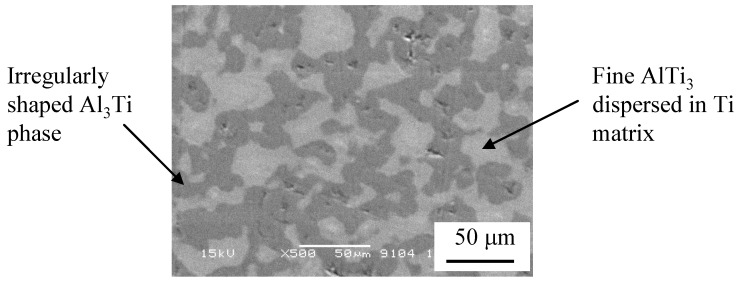
Scanning Electron Microcopy (SEM) images demonstrating the microstructure close to the tip of the specimen processed at 1350 °C [21].

**Figure 12:**
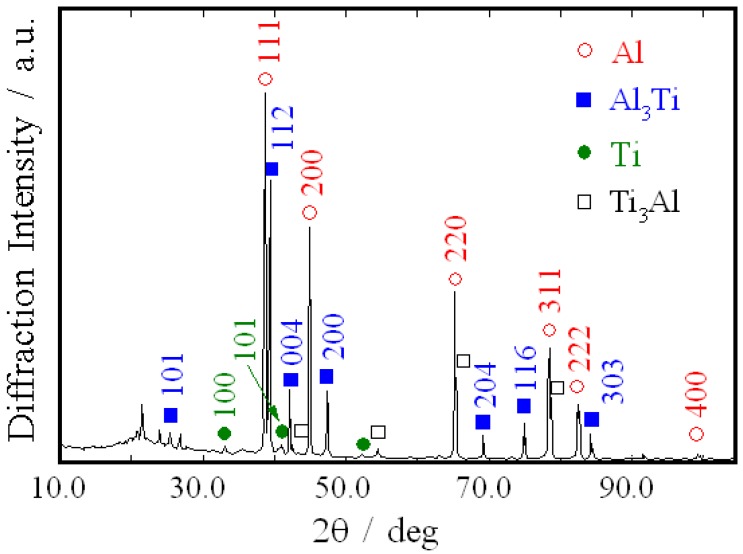
X-ray diffraction pattern of the sample processed at 1350 °C shown in Figure 11 [21].

The microstructure of RCMPM-FGMs close to the sample tip is presented in Figure 11. The structure of this part include irregularly shaped Al_3_Ti phase and very fine Ti_3_Al particles distributed in a Ti matrix. The evidence of this obtained structure is shown in the X-ray diffraction (XRD) pattern (Figure 12) of the sample processed at 1350 °C. From this figure, the high Al_3_Ti peaks can be observed along with small Ti_3_Al peaks thus proving the formation of Ti_3_Al intermetallics compound as well as Al_3_Ti at the processing temperature using our developed method. 

It was observed that Ti_3_Al intermetallics compound could be formed close to the FGM sample tip, where the Al melt temperature is lower than the rest of the sample due to the heat loss in the surroundings. It was reported that both Al_3_Ti and Ti_3_Al can be described as one-dimensional antiphase domain structures (1d-APS) [28]. Al_3_Ti has the D0_22_ structure, which is tetragonal with space group of I 4/m^3^ and the lattice parameters of the D0_22_ are; a = 3.84 Å and c = 8.59 Å [29]. On the other hand, Ti_3_Al has a D0_19_ hexagonal structure of a = 5.614 Å and c = 4.665 Å [29]. Following the reported experimental research and theoretical calculations [30,31], it is supposed that the formation of the Ti_3_Al-like (1d-APS) structure is both kinetically and thermodynamically simple and preferred relatively to the other Al-Ti rich phases. In a study by Wen *et al.* [32], both α_2_-Ti_3_Al and γ-TiAl phases could be obtained during semisolid forming of Ti and Al powders. The formation of such compounds was dependent on both the time and temperature of the alloying heat treatments. Furthermore, it has been reported that, if the Al-rich Al-Ti alloys are in non-equilibrium conditions (rapid quenching, severe mechanical treatment, *etc.*), the 1d-APSs might be formed first before the stable thermodynamic phases [33]. Since in RCMPM, there is a particular increase of the thermodynamic potential by centrifugal force [34], such that the maximum pressure exerted by the centrifugal force will be on the flask bottom (sample tip) while the minimum pressure will be in the bottom, then the thermodynamic conditions will be different between the two parts. Consequently, compounds like AlTi_3_ could be formed close to the sample tip. 

**Figure 13:**
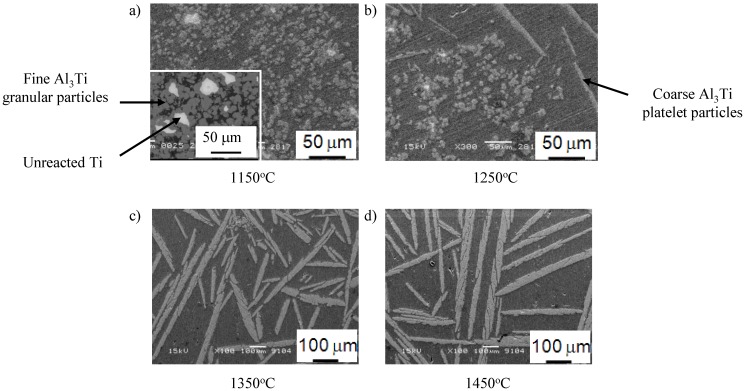
Scanning Electron Microcopy (SEM) micrographs of RCMPM-FGMs processed at different temperatures [21].

Figure 13 (a), (b), (c) and (d) show the scanning electron microscopy (SEM) micrographs of FGMs processed under different temperatures at 10 mm away from the tip. At relatively low temperature ~1150 °C, microstructure of the fabricated sample contains fine granular particles as observed in Figure 13 (a). Upon increasing the melting temperature to 1250 °C, the fine granular Al_3_Ti particles were first formed, then the coarse platelet-shaped Al_3_Ti particles could be obtained next to it, as shown in Figure 13 (b). The presence of these platelet-shaped particles next to the fine granular one, can be described as follows: First the Al melt penetrates into the Ti powder and the Al_3_Ti granular particles are formed. The heat of formation for Al_3_Ti at 25 °C is −35.6 kJ/g atom [22] and the corresponding adiabatic temperature rise is approximated to be ~1127 °C by taking the heat capacity as ~25 J/(K mol) [22]. This heat release due to the exothermic reaction can raise the temperature and accelerate the Al/Ti reaction in the following regions [35]. As a result, coarse Al_3_Ti platelets can be formed and then placed next to the fine particles under the action of the centrifugal force. At 1350 °C, as shown in Figure 13 (c), the typical Al/Al_3_Ti FGM containing platelet Al_3_Ti particles could be obtained. A further increase of the RCMPM temperature did not show a remarkable change in the microstructure, except for the coarser Al_3_Ti platelet particles observed for the FGM sample fabricated at 1450 °C, as presented in Figure 13 (d). 

**Figure 14:**
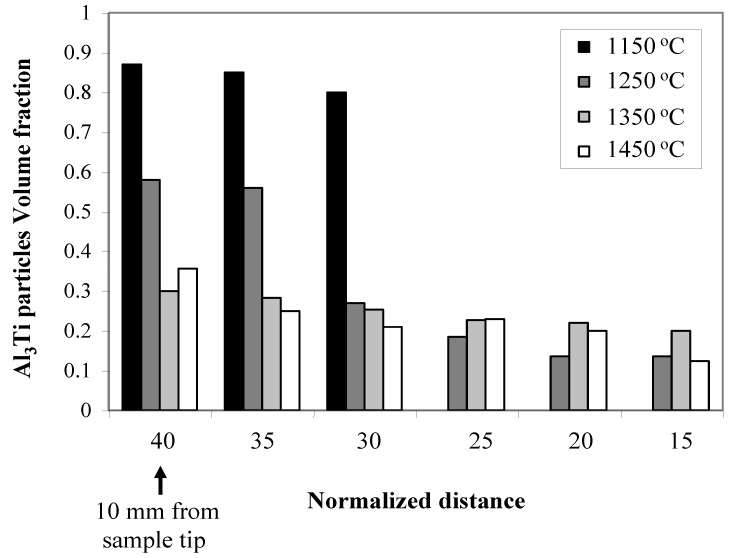
Al_3_Ti particles volume fraction distribution at different processing temperatures [21].

The centrifugal casting processing temperature in some reported studies showed a remarkable effect on the Al_3_Ti particles distribution in the fabricated Al-Al_3_Ti FGMs [15]. In RCMPM specifically, the processing temperature of the Al-Al_3_Ti/Ti_3_Al RCMPM-FGMs showed a strong influence not only on the Al_3_Ti particles shape but also on the distribution of Al_3_Ti particles size and volume fraction. Figure 14 shows the Al_3_Ti particles volume fraction distribution in the fabricated samples starting at 10 mm from the tip (where Al_3_Ti is present in a defined form) and up to 35 mm of its length. As previously described, only the Al_3_Ti fine granular particles were observed in the 1150 °C sample. These particles were distributed in 10 mm of the length and the rest of the sample was pure Al. This particle distribution is considered steep considering the sample length (50 mm). When the Al melt temperature increased to 1250 °C, the thickness of this layer containing the granular particles decreased and some platelet shaped Al_3_Ti particles could be obtained up to the sample end, as obvious from Figure 13 (b). This can be clearly understood from the slower volume fraction distribution of the sample fabricated at 1250 °C, shown in Figure 14, compared to that of 1150 °C. 

**Figure 15:**
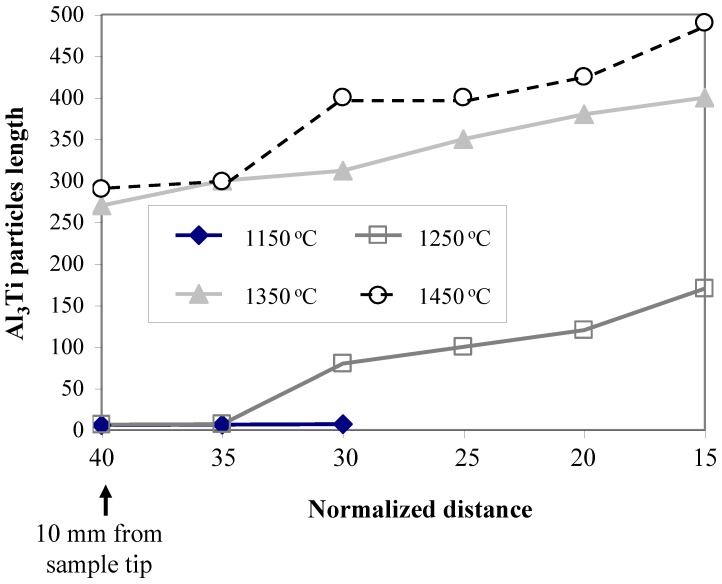
Distribution of Al_3_Ti particles length at different casting temperatures [21].

When increasing the processing temperature, the distribution gradient of Al_3_Ti platelet particles in these high temperature samples becomes broader from the sample tip up to end. In addition, the increase in the particles length when changed from granular to platelet morphology was further confirmed by the Al_3_Ti particles length distribution shown in Figure 15. Upon further heating of the Al melt up to 1350 °C, the coarse particles occurred more frequently and the granular morphology was rarely observed. Increasing the temperature up to 1450 °C resulted in absence of these low temperature fine particles and only coarse Al_3_Ti platelet particles could be found up to the end of the FGM sample as shown in Figure 15. The slower particle distribution gradient observed at higher temperatures is in accordance with the reported work of the Al-Al_3_Ti FGMs fabricated by CISM [15]. 

The formation of graded composition in the current FGMs samples can occur through two mechanisms. At 1150 °C, which is just below the liquidus of the master alloy (1153 °C), the centrifugal process is considered CSPM, in this case the formation of graded composition is believed to occur due to the density difference between Al_3_Ti and liquid Al. However, upon increasing the processing temperature, another mechanism is suggested [15]. This is the formation mechanism of the graded composition in case of A-B alloy *in situ* FGM under the centrifugal force, which can be summarized according to Watanabe and Oike [11] as follows:

1. Due to the density difference, partial separation of Al (2.261 Mg/m^3^ at 1100 °C) and Ti (4.1 Mg/m^3^ at 1680 °C) elements in the liquid state occur.
2. A chemical composition gradient is formed before the crystallization of the primary crystal.
3. The primary crystal in the matrix appears to depend on local chemical composition.
4. The primary crystal migrates according to density difference, and a further compositional gradient is formed.

Concluding, the Al3Ti compound formation depends on the processing temperature, so its migration and distribution would be temperature dependent processes as well.

### 4.1. Hardness distribution 

**Figure 16:**
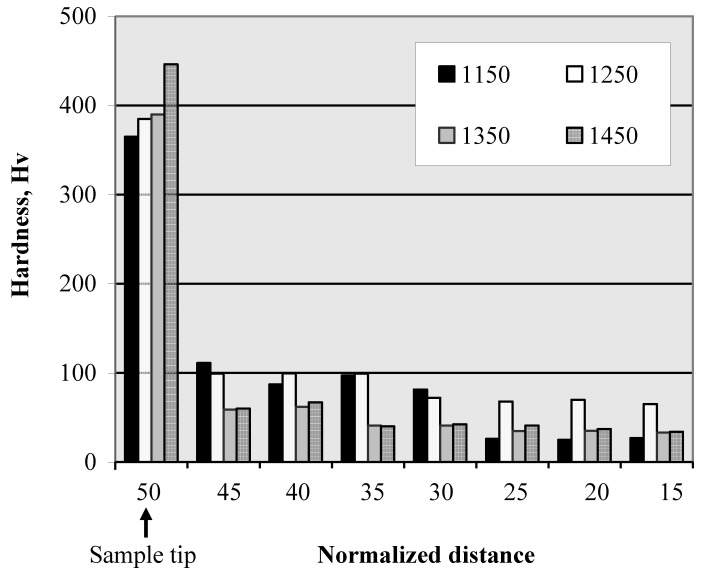
Vickers hardness distribution from the sample tip up to 35 mm of its length [21].

Figure 16 shows the hardness distribution from the sample tip and up to 35 mm of its length. In this figure, it is clear that the sample hardness at the tip is very high compared to the rest of it. This result can be due to the presence of very fine Ti_3_Al particles in a Ti matrix which consequently enhanced the hardness at the sample tip. This, in turn, resulted in a steep distribution of the hardness at all the processing temperatures. Following this, the fine granular Al_3_Ti particles shown in Figure 15 were present at 1150 °C and 1250 °C casting temperatures. The hardness of the samples containing such fine particles ranged between (78–110 Hv) which is significantly higher than the reported hardness of the Al/Al_3_Ti FGMs [29]. This is due to the presence of Al_3_Ti intermetallics as fine granular particles compared to the reported coarse Al_3_Ti platelets [5]. Once the Al_3_Ti particles formed with its platelet shape, the hardness of 1250 °C samples decreased again up to the sample end. When the casting temperature increased to 1350 °C and 1450 °C, the coarse Al_3_Ti particles formed and lower hardness values have been observed. The gradient distribution of the hardness in the fabricated FGMs is in accordance with the volume fraction distribution of Figure 14 and the particles size gradient shown in Figure 15.

A slower hardness distribution has been reported for Al-Al_3_Ti FGM rings fabricated by the CISM [25]. This is due to the gentle distribution of Al_3_Ti particles from the sample outer surface and up to its interior in case of CISM [11,15]. In our previous investigation [25], the Al-Al_3_Ti FGMs have been fabricated from an Al-5 mass% Ti ingot, therefore the hardness distribution was related only to the particles volume fraction. However, in the RCMPM experiments [21] the FGMs have been made using Ti powder and Al melt, thus formation of Al_3_Ti intermetallics occurred during the casting process. In summary, there are three parameters that can influence the hardness distribution in the Al‑Al_3_Ti/Ti_3_Al RCMPM-FGMs: formation of Al_3_Ti and/or Ti_3_Al particles, the particles characteristics (shape and size), and the type of particles distribution gradient at the processing temperature. 

## 5. Conclusions

In the current work, the fabrication of Al-Al_3_Ti/Ti_3_Al FGMs by a novel reaction centrifugal mixed‑powder method is reviewed. RCMPM showed a slower particles distribution at higher casting temperatures. Based on the obtained results; formation of Al_3_Ti intermetallics and its morphology in the prepared RCMPM-FGMs significantly change with the processing temperature. At relatively lower temperatures (1150–1250 °C), fine granular Al_3_Ti particles, Ti_3_Al intermetallics compound and un‑reacted Ti phase are observed. These granular Al_3_Ti particles could not be found at higher processing temperatures (1350 °C), where only coarse Al_3_Ti platelet particles were obtained. Casting at a higher temperature (1450 °C) resulted in more coarsening of the Al_3_Ti platelets. The hardness distribution in the fabricated FGMs showed a clear dependency on the particles type, their size, and their distribution at the processing temperature.
